# *Materials* Best Paper Award 2015

**DOI:** 10.3390/ma8020829

**Published:** 2015-02-17

**Authors:** Maryam Tabrizian

**Affiliations:** Department of Biomedical Engineering, Faculty of Medicine/Faculty of Dentistry, Duff Medical Science Building, McGill University, 3775 University Street, Montreal, QC H3A 2B4, Canada; E-Mail: maryam.tabrizian@mcgill.ca

*Materials* has established an annual award for the best article and for the best review published in *Materials* in order to acknowledge the outstanding contributions of our authors in the area of materials science and engineering.

Papers were selected by the Editors-in-Chief of *Materials* from among all articles and reviews published in 2011. We are now pleased to announce the five winners for the second edition of “*Materials* Best Paper Award” for 2015:

**Article Award:**
  First Prize
    **Nieves Espinosa, Henrik Friis Dam, David M. Tanenbaum, Jens W. Andreasen, Mikkel Jørgensen and Frederik C. Krebs**
    Roll-to-Roll Processing of Inverted Polymer Solar Cells using Hydrated Vanadium(V)Oxide as a PEDOT:PSS Replacement
    *Materials* **2011**, *4*(1), 169–182; doi:10.3390/ma4010169
    Available online: http://www.mdpi.com/1996-1944/4/1/169
  Second Prize
    **Shingo Tachikawa, Atsushi Noguchi, Takeharu Tsuge, Masahiko Hara, Osamu Odawara and Hiroyuki Wada**
    Optical Properties of ZnO Nanoparticles Capped with Polymers
    *Materials* **2011**, *4*(6), 1132–1143; doi:10.3390/ma4061132
    Available online: http://www.mdpi.com/1996-1944/4/6/1132
  Third Prize
    **Luigi Russo, Francesco Colangelo, Raffaele Cioffi, Ilaria Rea and Luca De Stefano**
    A Mechanochemical Approach to Porous Silicon Nanoparticles Fabrication
    *Materials* **2011**, *4*(6), 1023–1033; doi:10.3390/ma4061023
    Available online: http://www.mdpi.com/1996-1944/4/6/1023
**Review Award:**
  First Prize
    **Kirill V. Axenov and Sabine Laschat**
    Thermotropic Ionic Liquid Crystals
    *Materials* **2011**, *4*(1), 206–259; doi:10.3390/ma4010206
    Available online: http://www.mdpi.com/1996-1944/4/1/206
  Second Prize
    **Gerrard Eddy Jai Poinern, Nurshahidah Ali and Derek Fawcett**
    Progress in Nano-Engineered Anodic Aluminum Oxide Membrane Development
    *Materials* **2011**, *4*(3), 487–526; doi:10.3390/ma4030487
    Available online: http://www.mdpi.com/1996-1944/4/3/487

The *Materials* journal team congratulates the authors of these five exceptional papers and acknowledges their valuable contributions to *Materials* and to our scientific community. In recognition of their accomplishments, Dr. Frederik C. Krebs, Dr. Hiroyuki Wada and Dr. Luca De Stefano, our awardees for their original contributions, will receive 600 CHF, 400 CHF and 200 CHF respectively. In addition, *Materials* will offer them the publication of a paper in *Materials* free of charge in open access format after the usual peer-review procedure. The publication of one research paper free of charge in *Materials* will also be offered to Dr. Sabine Laschat and Dr. Gerrard Eddy Jai Poinern, the winners of the Best Review Papers award.

Prize Awarding Committee

Editor-in-Chief

**Prof. Dr. Maryam Tabrizian**

Department of Biomedical Engineering, Faculty of Medicine/Faculty of Dentistry, Duff Medical Science Building, McGill University, 3775 University Street, Montreal, QC H3A 2B4, Canada; E-Mail: maryam.tabrizian@mcgill.ca

*Editor-in-Chief*, Section “Biomaterials”

**Prof. Dr. Aldo R. Boccaccini**

Institute of Biomaterials, Department of Materials Science and Engineering, University of Erlangen-Nuremberg, 91058 Erlangen, Germany

E-Mail: aldo.boccaccini@ww.uni-erlangen.de

*Editor-in-Chief*, Section “Advanced Composites”

**Prof. Dr. Luciano Feo**

Department of Civil Engineering and Nano_Mates Center, University of Salerno, 84084 Fisciano (SA), Italy

E-Mail: l.feo@unisa.it

*Editor-in-Chief*, Section “Structure Analysis and Characterization”

**Prof. Dr. Jung Ho Je**

Department of Materials Science and Engineering, Pohang University of Science and Technology, San 31 Hyojadong, Pohang 790-784, Korea

E-Mail: jhje@postech.ac.kr

*Editor-in-Chief*, Section “Porous Materials”

**Prof. Dr. Rafael Luque**

Departamento de Quimica Organica, Universidad de Cordoba, Campus de Rabanales, Edificio C-3 (Marie Curie-Anexo), Carretera Nacional IV-A, Km. 396, 14014 Cordoba, Spain

E-Mail: q62alsor@uco.es

